# Basophilic Stippling Unmasks Pyrimidine 5′‐Nucleotidase Deficiency in a G6PD‐Deficient Patient

**DOI:** 10.1002/ajh.70495

**Published:** 2026-09-10

**Authors:** Victor Bobée, Stéphane Moutereau, Maïssa Souissi, Pierre Lebreton, Loïc Garcon

**Affiliations:** ^1^ Department of Laboratory Hematology Rouen University Hospital Rouen France; ^2^ Department of Clinical Biology Centre Henri Becquerel Rouen France; ^3^ Département de Biochimie‐Biologie Moléculaire‐Pharmacologie‐Génétique Médicale Henri‐Mondor University Hospital‐ UPEC Créteil France; ^4^ Hematology Department Groupe Hospitalier du Havre Le Havre France; ^5^ Service d'Hématologie Biologique, CHU Amiens Picardie Amiens France

**Keywords:** anemia, enzymopathy, G6PD deficiency, hemolysis, P5N

A 30‐year‐old man was referred to the hematology department for evaluation of chronic hemolytic anemia. His medical history included glucose‐6‐phosphate dehydrogenase (G6PD) deficiency, diagnosed during childhood following an episode of acute anemia that required red blood cell transfusion, and the ongoing hemolysis had long been attributed to this known enzymatic defect. No neonatal jaundice was reported. However, the patient described life‐long jaundice and intermittent episodes of dark urine, without dyspnea. Physical examination showed splenomegaly. Initial blood tests showed moderate macrocytic anemia (hemoglobin 100 g/L, MCV 101 fL) with marked reticulocytosis (283 × 10^9^/L). Biochemical evidence of hemolysis included undetectable haptoglobin, elevated LDH (1064 IU/L), and total bilirubin of 66 μmol/L. Peripheral blood smear examination revealed polychromasia and numerous basophilic stipplings (Figure 1A,B). A supravital stain with brilliant cresyl blue demonstrated Heinz bodies within erythrocytes, indicating oxidized and denatured hemoglobin (Figure 1C). To further investigate the chronic hemolysis, a comprehensive workup was conducted, including the eosin‐5‐maleimide binding test, ektacytometry, hemoglobin electrophoresis, direct antiglobulin test, and assays for G6PD and pyruvate kinase activity. All results were normal except for markedly decreased G6PD activity, consistent with the known deficiency. Due to the presence of prominent basophilic stippling, additional erythrocyte enzyme assays were performed and pyrimidine 5′‐nucleotidase (P5N) activity was profoundly reduced (< 1 IU/g Hb, normal range 5.6–9.0), while glucose phosphate isomerase (GPI), triosephosphate isomerase (TPI), and hexokinase (HK) levels were normal. These findings led to the diagnosis of P5N deficiency in addition to the pre‐existing G6PD deficiency. Folic acid supplementation was initiated, and the patient was enrolled in long‐term follow‐up every 3 years to monitor for potential iron overload.

**FIGURE 1:**
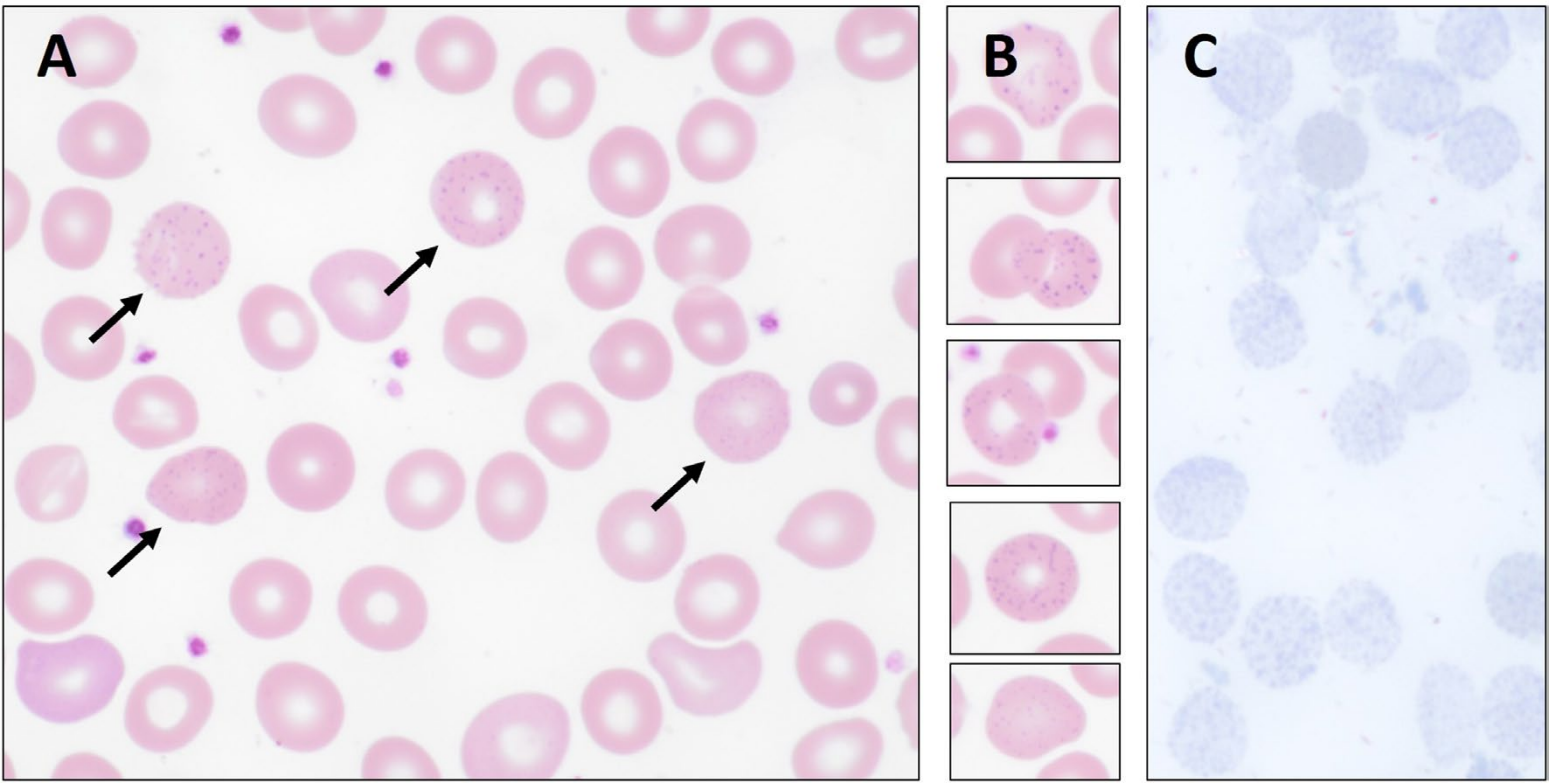
(A) Peripheral blood smear showing numerous red blood cells with basophilic stippling (arrows), May‐Grünwald Giemsa stain, original magnification ×1000. (B) Insets highlighting red blood cells with prominent basophilic inclusions. (C) Supravital staining with brilliant cresyl blue reveals multiple Heinz bodies within red blood cells, representing denatured, oxidized hemoglobin aggregates. Original magnification ×1000.

Patients with G6PD deficiency are typically asymptomatic between hemolytic episodes, except in rare cases involving class A variants according to the WHO classification [1]. Heinz bodies, representing denatured oxidized hemoglobin, are classically observed in G6PD deficiency and in unstable hemoglobinopathies. Unlike G6PD, P5N is an enzyme involved in the degradation of pyrimidine nucleotides. Its deficiency is a rare congenital enzymopathy that leads to the accumulation of pyrimidine derivatives in erythrocytes, forming insoluble aggregates responsible for basophilic stippling and resulting in chronic hemolytic anemia [2]. While basophilic stippling is not specific to P5N deficiency and may also be seen in conditions such as lead poisoning or myelodysplastic syndromes, it remains a hallmark feature that can strongly suggest the diagnosis. In this case, the unusually dense and widespread basophilic inclusions prompted further enzymatic investigation, leading to the diagnosis. Importantly, the co‐existence of G6PD deficiency may have contributed to increased red blood cell fragility, thereby exacerbating the hemolytic phenotype of the P5N deficiency. This case illustrates the importance of investigating alternative or coexisting etiologies in patients with G6PD deficiency who present with chronic hemolysis. It also underscores the importance of meticulous blood smear examination by experienced personnel in guiding the diagnostic process and contributes to the limited literature on the exceptional coexistence of G6PD and P5N deficiencies.

## Author Contributions

V.B. analyzed data and wrote the paper, S.M., M.S., P.L. analyzed data, L.G. coordinated the study and supervised analysis.

## Funding

The authors have nothing to report.

## Disclosure

All data and images presented in this report have been fully anonymized, and no information allowing identification of the patient is disclosed.

## Ethics Statement

This report is based on retrospective review of clinical and laboratory data collected during routine patient care. All data were fully anonymized prior to publication and handled in accordance with applicable French regulations and institutional policies.

## Conflicts of Interest

The authors declare no conflicts of interest.

## Data Availability

All data are available from the corresponding author upon reasonable request.
